# Supplement to Ziemssen’s Cyclopedia of the Practice of Medicine

**Published:** 1881-07

**Authors:** 


### BIBLIOGRAPHICAL.

Supplement to Ziemssen's Cyclopedia of the Practice of Medicine. Edited by Geo L. Peabody, M.D., Instructor in Pathology and Practice of Medicine, College of Physicians and Surgeons, New York, etc. Wm. Wood & Co., N. Y. 1881.

This volume, of like binding and print to Ziemssen's publications, is intended as an index of the improvements and new discoveries that have taken place since the first few volumes of the Clyclopedia were published. The scope of the work is extended, as the five or six years that have elapsed since the publication of the first series of Ziemssen's, have been marked by varied and important advances on the subjects therein discussed.

The corps of contributors to this work is composed of authors of the first standing, and the materials have been derived from the literature of all countries. Much work has been found necessary to bring the book up to the standard required to make it a correct and perfect compend of the important advances that have occurred for the last few years in the practice of medicine, and it is believed that the effort made in this direction has been sufficiently successful to commend the work to the favorable consideration of the profession. Among the more important subjects treated of may be mentioned typhoid fever, yellow fever, croup and diphtheria, malarial diseases, cerebro-spinal meningitis, syphilis, diseases of the nose, lungs, heart, bladder, urethra, etc., vaso-motor and trophic neuroses, toxicology, and so forth.

Altogether it is a very interesting volume, and much important information may be gained from its perusal.

